# Anticolonization of Carbapenem-Resistant *Klebsiella pneumoniae* by *Lactobacillus plantarum* LP1812 Through Accumulated Acetic Acid in Mice Intestinal

**DOI:** 10.3389/fcimb.2021.804253

**Published:** 2021-12-15

**Authors:** Rushuang Yan, Ye Lu, Xiaoqing Wu, Peihao Yu, Peng Lan, Xueqing Wu, Yan Jiang, Qi Li, Xionge Pi, Wei Liu, Jiancang Zhou, Yunsong Yu

**Affiliations:** ^1^ Department of Critical Care Medicine, Sir Run Run Shaw Hospital, Zhejiang University School of Medicine, Hangzhou, China; ^2^ Key Laboratory of Microbial Technology and Bioinformatics of Zhejiang Province, Sir Run Run Shaw Hospital, Hangzhou, China; ^3^ Department of Rehabilitation, Sir Run Run Shaw Hospital, Zhejiang University School of Medicine, Hangzhou, China; ^4^ Department of Infectious Diseases, Sir Run Run Shaw Hospital, Zhejiang University School of Medicine, Hangzhou, China; ^5^ Department of Emergency Medicine, Lanxi People’s Hospital, Lanxi, China; ^6^ Institute of Plant Protection and Microbiology, Zhejiang Academy of Agricultural Sciences, Hangzhou, China

**Keywords:** carbapenem-resistant, *Klebsiella pneumoniae*, *Lactobacillus plantarum*, anticolonization, Acetic acid

## Abstract

Carbapenem-resistant *Klebsiella pneumoniae* (CRKP) is highly prevalent and poses a significant threat to public health. In critically ill patients, gut colonization is considered to be the reservoir of recurrent CRKP infection. Therefore, eliminating CRKP carriage in the intestine is critical for preventing subsequent CRKP infection. In the present study, *Lactobacillus plantarum* LP1812, a probiotic that can inhibit CRKP *in vitro*, was used as a candidate probiotic to investigate its efficacy for CRKP anticolonization. Compared with the control, mice fed with 1×10 ^8^ CFU *L. plantarum* LP1812 exhibited significant CRKP clearance from 1×10 ^4^ CFU/mg to less than 10 CFU/mg in mice feces. Furthermore, 16S RNA gene sequencing revealed that *L. plantarum* LP1812 modulated mice microbiota by increasing the relative abundance of the genus *Halomanas*, *Blautia*, and *Holdemania*. Further KEGG pathway enrichment analysis revealed that fatty acid-utilizing bacteria, such as acetate-producing *Bacteroidetes* and *Blautia* flourished in mice fed with *L. plantarum* LP1812. Moreover, we found that the concentration of acetic acid was higher in *L. plantarum* LP1812, which inhibited the growth of *K. pneumoniae* strains *in vitro*. Meanwhile, mice intragastrically administered with acetic acid exhibited significantly increased CRKP elimination *in vivo*. In conclusion, *L. plantarum* LP1812 is a potential candidate for intestinal CRKP anticolonization by regulating the intestinal microbiota and inhibiting CRKP *via* increased acetic acid in the intestinal lumen.

## Introduction

Carbapenem-resistant *Klebsiella pneumoniae* (CRKP) is an increasingly common nosocomial pathogen that causes infection in a variety of locations, including the lower respiratory tract, urinary tract, and the bloodstream (Pendleton et al., 2013), posing a significant threat to public health (Michalopoulos et al., 2011; Musicha et al., 2017). Among other infections, CRKP bacteremia is hazardous for having a high all-cause mortality rate of up to 40% (Kontopoulou et al., 2019; Wang et al., 2019). Notably, patients with CRKP gut colonization have a greater risk of bloodstream infection and recurrent infection (Kontopoulou et al., 2019; Wang et al., 2019). Therefore, eliminating intestinal carriage of CRKP and thus reducing secondary infection is an intuitively appealing concept.

Numerous decolonization studies have been undertaken, including selective digestive decontamination with colistin or aminoglycosides (Machuca et al., 2016; Stoma et al., 2018), custom-made bacteriophage (Hua et al., 2017; Corbellino et al., 2020; Liu et al., 2021) and fecal microbiota transplantation (FMT) (Alagna et al., 2020; Gouveia et al., 2020; Ueckermann, et al., 2020). Although those methods demonstrated some decolonization effects, the possibility of drug resistance induced by gentamycin or colistin, the high cost and prolonged waiting time for phage, and the possibility of infectious agent transmission accompanied by FMT rendered them impractical for generalization. Recent advances in pathogen decolonization have included the use of novel treatments that are microbiome-based or microbiome-targeted (Mullineaux-Sanders et al., 2018; Kontopoulou et al., 2019). Using these methods, two patients with *Clostridioides difficile* infection were successfully treated with 33 commensal isolates (Petrof et al., 2013). Araos et al. (2016) reported that patients with a higher abundance of *Lactobacillus* spp. were less likely to be colonized with antibiotic-resistant bacteria during hospitalization. Therefore, probiotics are promising candidates for eradicating CRKP in the intestine with fewer adverse effects and expenses.

As a common probiotic, *Lactobacillus* spp. has been largely recognized as safe for the human body, and its anti-pathogen effect has been confirmed (Ripamonti et al., 2011). However, the exact mechanisms of action of *Lactobacillus* spp. are equivocal. In this study, we established that *Lactobacillus plantarum* LP1812 can improve mammalian gut health by increasing intestinal fatty acid-utilizing bacteria such as *Bacteroidetes* and *Blautia*. In addition to the strong CRKP elimination and killing effect of *Bacteroidetes* and *L. plantarum per se*, abundant acetic acid secreted by *L. plantarum* can acidify the CRKP intracellular environment and inhibit its growth. These synergistic effects result in efficient intestinal CRKP clearance.

## Materials and Methods

### Bacteria Strains and Culture Conditions

The four *Klebsiella pneumoniae* strains: K. pneumoniae ATCC1705, NTUH-K2044, ZKP25 (carbapenem-resistant) and ZKP16 (carbapenem sensitive) were obtained from Sir Run Run Shaw Hospital, Zhejiang University School of Medicine. MRS (de Man, Rogosa & Sharpe), and LB were used as basic growth media for *Lactobacilli* spp. and *K. pneumoniae*, respectively. All strains were frozen at -80°C in 20% glycerin bouillon. Pathogen resuscitation was performed by inoculating them on MH (Mueller-Hinton) agar at 37°C overnight.

### Co-Culture Assays

*K. pneumoniae* and *Lactobacilli* spp. strains were revived on MH agar, then cultured in their basic broth with a single colon and shook overnight. Subsequently, 50 μL *K. pneumoniae* culture liquid was incubated in MRS broth as a blank control, while the test groups were incubated in modified MRS broth containing 10% overnight *Lactobacilli* spp. suspension obtained by filtering *Lactobacilli* spp. culture liquid using a 0.45 μM membrane. After that, the mixtures were shaken anaerobically for 12 hours at 37°C. Every 2 hours, sterile phosphate-buffered saline (PBS) was added to the co-culture system, and the serial dilutions were plated on ampicillin 0.1 mg/mL MH screening agar. The exact number of *K. pneumoniae* colonies at each time point was determined the next day. All experiments were repeated three times.

### Growth Curve

After reviving and filtering the four *Lactobacilli* spp. strains, the supernatant was collected and mixed with LB broth into different concentrations (5%, 10%, 15%, and 20%). Additionally, modified LB broth was prepared by combining it with glacial acetic acid and HCl to ensure that each liter LB broth contained 50 mmol acetates and had a final pH of 3. The modified LB was added to normal LB at 5%, 10%, 20%, and 50% concentrations. Subsequently, 10μL of overnight *K. pneumoniae* was inoculated into 1 mL of a separate culture broth. The blank controls were MRS and 0% LB in different batch assays. Bioscreen C was used to detect the OD_600_ value of samples at different time points (every five minutes) and BioScreener was used to collect and store data.

### Murine CRKP Colonization Model

We initially prepared the cell concentration of ZKP25 and LP1812 overnight bacterial culture fluid, which was subsequently used for dilutions to obtain the final concentration. Mice were purchased from Zhejiang University’s Institutional Animal Ethics Committee with the number ZJU20160154. All BALC/c mice were female and weighed 20-25 g, when purchased from Ziyuan, Hangzhou, China.

**Figure 1:**
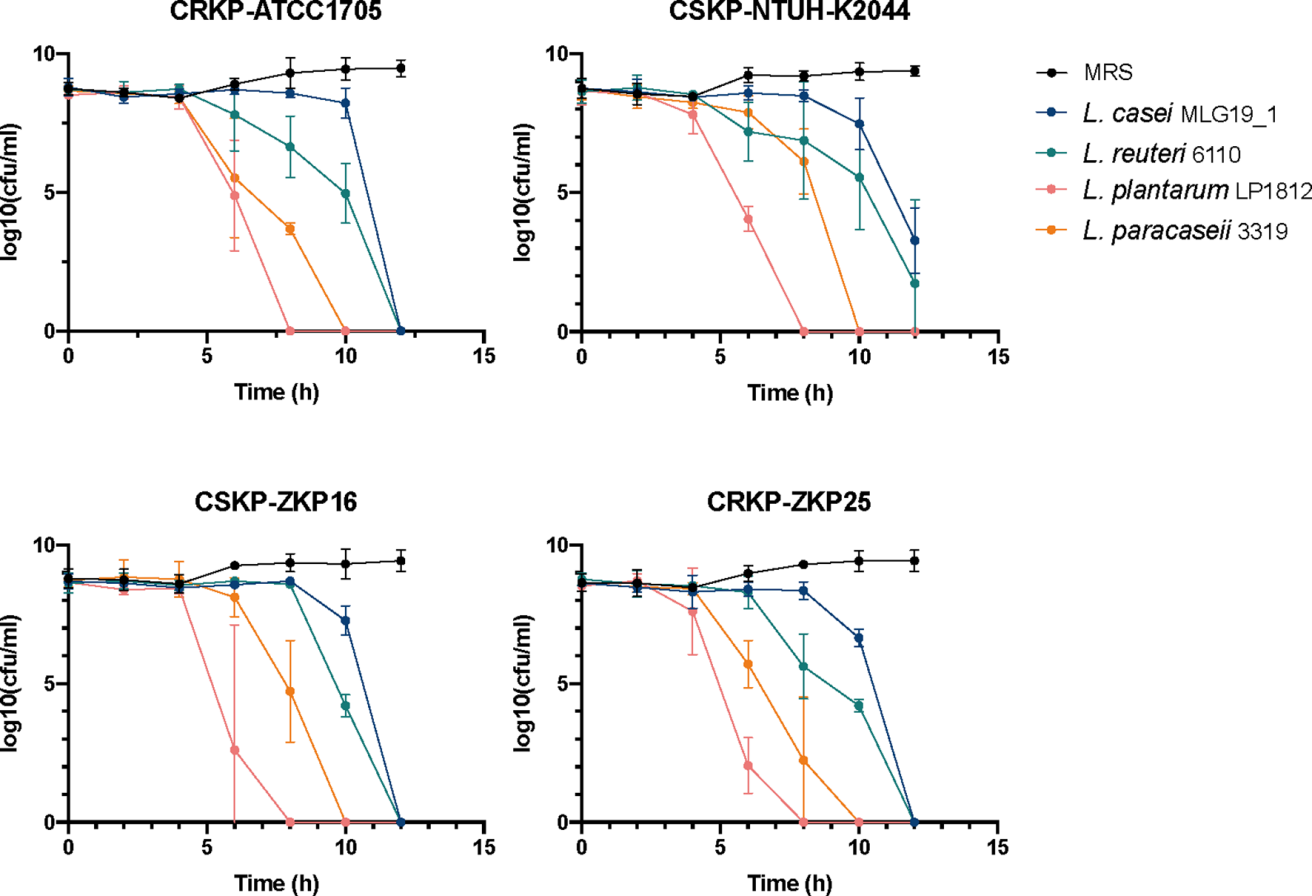
The effects of co-culture trails of *K. pneumoniae.* and *Lactobacillus* spp. NTUH-K2044, a classical hypervirulent *K. pneumoniae* isolate, ATCC1705, a standard carbapenem-resistant *K. pneumoniae* isolate, ZKP16, a carbapenem-sensitive clinical isolate, ZKP25, a carbapenem-resistant clinical isolate.

**Figure 1A** shows the procedure for establishing the CRKP intestinal colonization model. In our set-up, we first cleared the animal gut from day -10 to day -4 using a broad-spectrum antibiotic combination, KGCVM, containing kanamycin 0.4 mg/mL, gentamycin 0.035 mg/mL, polymyxin B 0.085 mg/mL, vancomycin 0.045 mg/mL, and metronidazole 0.215 mg/mL (Chen et al., 2008). Subsequently, mice in each group were gavaged with different treatments for three days (day-3 to day-1). Mice in the L.P. group were treated with 200 μL 5×10^8^ colony-forming units (CFU) per milliliter bacterial suspension, while mice in the FMT group were treated with 200 μL fecal suspension. Suspensions of *L. plantarum* and feces were combined in a 1:1 ratio, and mice in the MIX group were gavaged with 200 μL. Additionally, all mice in the Blank group were given 200 μL PBS, as a negative control. Animals in four groups were all orally inoculated with about 200 μL ZKP25 (5×10^7^ CFU/mL) on day 0. The CFU of *K. pneumoniae* strains in mice intestines was reflected by culturing mouse feces samples on-screen plants MEM 8 (meropenem 8 μg/mL). The mouse feces were collected on days 1, 2, 3, 5, and 7, then weighted and mixed with 1 mL sterile PBS, diluted ten times, and inoculated on MH screening agar containing 8 μg/mL meropenem. Parts of the collected feces were stored at -80°C for subsequent microbiome sequencing.

Blank group mice were given normal LB broth instead of PBS in subsequent CRKP anti-colonization assays (**Figures 5** and **S3D**). In addition, mice in the acetic acid group were gavaged with a modified LB broth that we had previously prepared. The acetic acid + FMT group was treated with a combination of modified LB and FMT suspension (1:1 ratio).

### 16S rRNA Sequencing and Analysis

DNA extraction was performed on 136 feces samples to obtain information on intestinal microbiota changes using the PowerSoil^®^ DNA Isolation Kit (MO BIO, Cat.No.12888). To amplify 16S rRNA genes from distinct regions, specific primers with barcodes were used, and PCR reactions were performed using Phusion^®^ High-Fidelity PCR Master Mix (New England Biolabs). TruSeq^®^ DNA PCR-Free Sample Preparation Kit (Illumina, USA) was used to generate the sequencing libraries, which were subsequently sequenced on an Ion S5TM platform to yield 400 bp/600 bp single-end reads. After analyzing sequences using the Uparse software (Uparse V8.1.1861), OTUs (Operational Taxonomic Units) were obtained. To obtain species information, the SILVA database was used as a reference to annotate representative sequences in OTUs. Multiple sequence alignments were performed using the MUSCLE software (Version 3.8.31) to determine the phylogenetic relationship of different OTUs and the differences in the dominant species in different samples (groups).

The LDA score was calculated using LEfSe after dividing all feces samples into two categories based on the OTUs table: KP free and KP high loads (the ratio of *K. pneumoniae* > 0.01). The aim was to identify specific enriched bacteria species in KP-free feces. Principal coordinate analyses (PCoA) in R software were used to determine the microbiota structure of different samples. Tax4Fun was used to estimate KEGG function and calculate group differences based on functional abundance among samples, and analyze bacterial function in various fecal samples.

### Evaluation of Intracellular pH

Initial preparation of the acetic acid modified LB solutions were required for the CRKP intracellular pH assays. The pH-matched LB broth was then prepared by combining varying amounts of HCl to the pH of 3, 4, 5, 6, 7, 8 as determined by pH strips. The ratio-metric dye BCECF-AM was added to the culture conditions, which were detected using an enzyme-labeled instrument. The ratio of fluorescence emission from 485 and 444 spectra was used to determine intracellular pH. To draw a standard curve for subsequent intracellular pH detection, 10 μL of overnight cultured CRKP strains were inoculated into 1 mL of the pH-matched LB mixed with 1 μM BCECF-AM and 50 μM CCCP (a cell membrane depolarizer). The emission ratio of BCECF-AM was monitored throughout a 2 h period at 37°C to allow the dye to equilibrate. The ratio of λ_em_ 485 to λ_em_ 444 was calculated and used to generate a standard curve. The ratio-metric measurements of BCECF-AM at different concentrations of acetic acid and extracellular pH were converted to absolute intracellular pH values using linear interpolation to the previously obtained standard curve measurements.

### Statistical Analysis

All statistical analyses were performed in R software (Version R 4.1.0), while ggplot2 was used to draw the graphs. In addition, we introduced python 2.7 to calculate LDA score (Segata et al., 2011).

## Results

### *Lactobacillus* spp. Can Kill *Klebsiella* spps. *In Vitro*

To determine the inhibitory effect of *Lactobacilli* spp. against *K. pneumoniae*, we respectively co-cultured four lactobacilli strains, *Lactobacillus casei* MLG19_1, *Lactobacillus reuteri* 6110, *Lactobacillus paracaseii* 3319 and *Lactobacillus plantarum* LP1812 with four different *K. pneumoniae* strains, which were classical hypervirulent *K. pneumoniae* NTUH-K2044, Carbapenem-sensitive *K. pneumoniae* (CSKP) ZKP16, and CRKP ATCC1705, clinical CRKP ZKP25 isolates (**Figure 1**). After 12 hours of co-incubation, the four probiotics had completely eradicated ZKP16 and two CRKP strains. In addition, *L. plantarum* LP1812 was able to eradicate *K. pneumoniae* strains in 8 hours.

**Figure 2:**
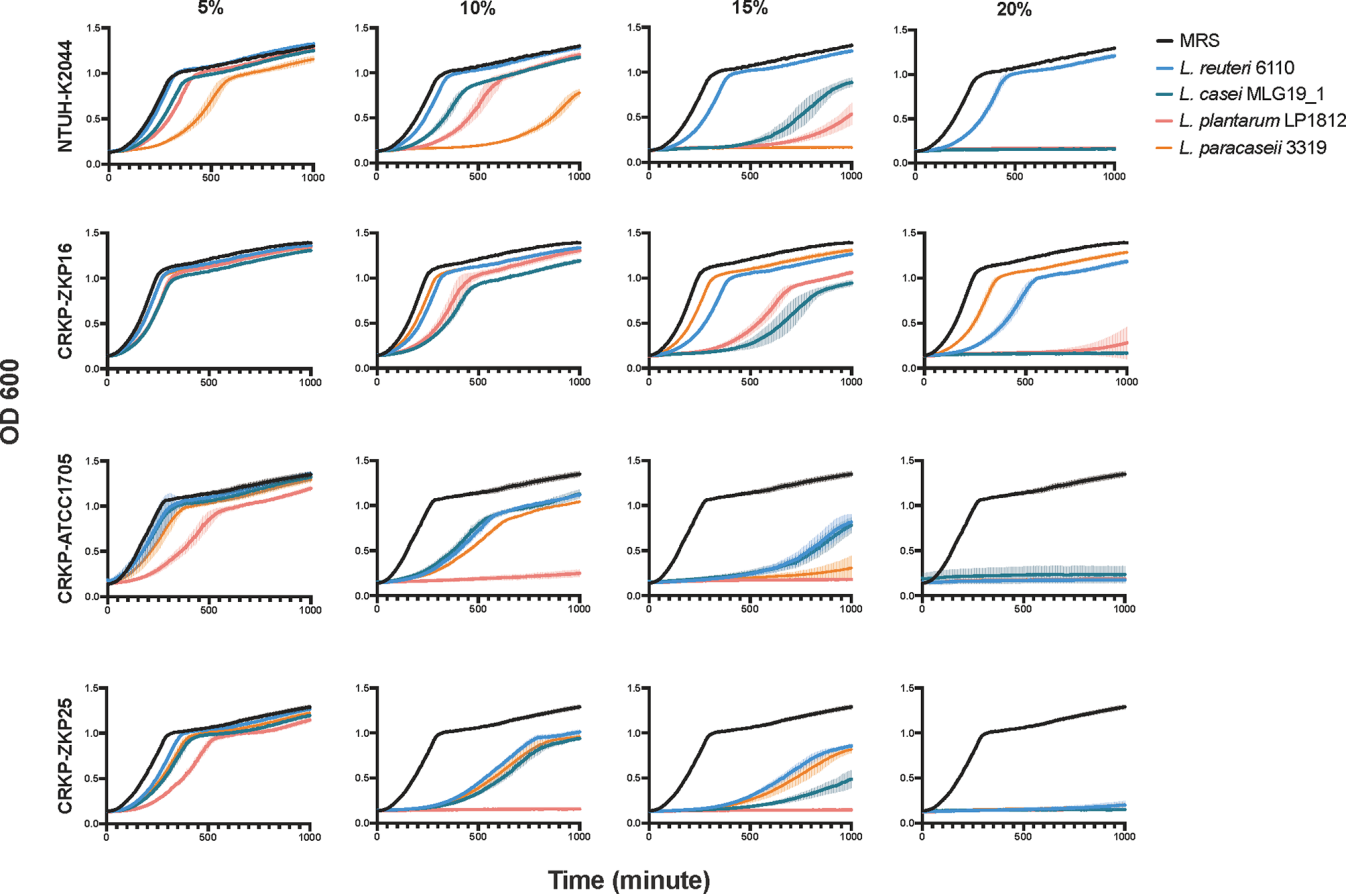
The growth curves of *K. pneumoniae* in *Lactobacillus* spp. supernatants of different species and concentrations. The amount of *K. pneumoniae* was exhibited by variations in absorption values at OD600.

To determine whether the above-mentioned pathogen-killing effects were attributable to the probiotics *per se* or certain elements in the supernatant, we performed growth curve assays between *K. pneumoniae* strains and the supernatant of four distinct *Lactobacillus* spp. at different concentrations. As shown in **Figure 2**, the supernatant of all four *Lactobacillus* spp. strains significantly inhibited *K. pneumoniae* growth at a 10% concentration, and completely inhibited its growth at 20%, except for hypervirulent *K. pneumoniae* NTUH-K2044 (**Figure 2**). Similar to the co-culture results, *L. plantarum* LP1812 had the highest inhibitory effect against *K. pneumoniae*, whereas *L. casei* MLG19_1 had the least.

### *L. plantarum* LP1812 Exhibits Anti-CRKP Colonization Ability *In Vivo*

**Figure 3:**
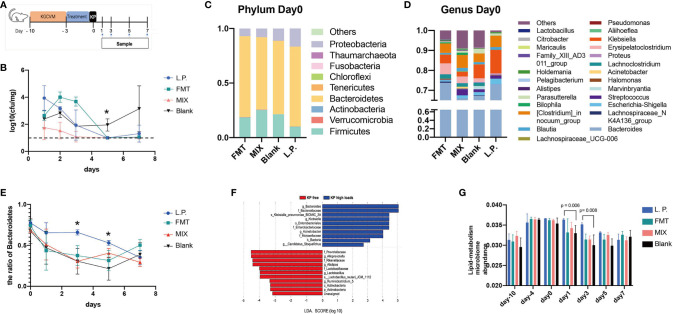
CRKP anticolonization experiments and 16S analysis. **(A)** Schedule design for animal trails. **(B)** The anti-CRKP assay findings. The Y-axis represents loads of CRKP strains in mouse feces (n = 4). The limit of detection is represented by the imaginary line. Blank represents the negative control, whereas FMT represents the positive control. L.P. means inoculating mice with *L. plantarum* LP1812, FMT stands for fecal microbiota transplantation, and MIX refers to pretreatment mice with mixtures of LP1812 and FMT suspensions. The percentages of intestinal microbiota community abundance at the Phylum **(C)** and Genus **(D)** levels. The relative abundances of various bacteria were calculated by averaging the data from five/four replicates within each group (FMT and Blank have four mice, L.P. and MIX have five mice) obtained from 16S OTU tables. **(E)** The ratio of *Bacteroidetes* in mouse intestinal microbiome calculated from the OTU tables. **(F)** LEfSe analysis between two groups that were re-classified in OTU tables based on loads of CRKP ZKP25. **(G)** The lipid metabolism relative microbiome ratios in feces microbiomes. The ratios were obtained from KEGG analysis. *denotes p-value < 0.05.

Because *Lactobacillus* spp. showed a significant anti-CRKP effect *in vitro*, we wondered if pre-treatment with *Lactobacillus* spp. strains could interfere with *K. pneumoniae* colonization. Therefore, we depleted the microbiota in adult mice using a broad spectrum KGCVM antibiotic combination and then pre-treated with different interventions before CRKP ZKP25 inoculation (**Figure 3A**). Given that FMT is commonly used in clinical practice to restore intestinal microbiota and consequently clear intestinal colonized pathogens (Liptak et al., 2021), it was considered as a positive control. Before ZKP25 inoculation, four interventions were administered, including Blank (control), FMT, *L. plantarum* LP1812 (L.P. group), and MIX (combined *L. plantarum* LP1812 with FMT). Mice pretreated with *L. plantarum* LP1812 showed comparable intestinal protectivity with FMT (**Figure 3B**), and the CRKP strains were below the limit of detection by day 5. Furthermore, when the *L. plantarum* LP1812 and FMT were combined, this obvious impact was amplified (MIX group).

### *L. plantarum* LP1812 Affects CRKP Colonization by Modulating Intestinal Microbiota in Mice

To investigate the changes in the microbiota of mouse feces following the aforementioned pretreatments, we sequenced the 16S rRNA of the collected feces and determined the microbial community composition in different groups. By day 5, the intestinal microbiota of the four groups was restored to normal (**Figure S1**). Pre-treatment with *L. plantarum* LP1812 was beneficial to mouse gut microbiota since both L.P. and MIX exhibited significantly higher α-diversity when compared with the Blank and FMT, respectively. Additionally, at phylum levels, *Bacteroidetes* dominated the mouse intestinal microbiota on day 0 (after three days of treatment), followed by *Firmicutes* and *Proteobacteria* (**Figure 3C**). Between day 0 and day 5, the L. P. group exhibited the highest *Bacteroidetes* abundance among the four groups (**Figure 3E**). After comparing the composition of the intestinal microbiota at the genus level (**Figure 3D**), we found that the abundance of beneficial bacteria such as *Halomanas, Blautia*, and *Holdemania* was increased in all three treatment groups, with the first being significantly higher when comparing L.P. and Blank (p-value = 0.010). In the Blank group, in contrast, *Erysipelatoclostridium* and *Citrobacter* were significantly increased (compared with the L.P. group, the p-value was 0.018, 0.002, and 0.030, respectively).

To investigate which bacteria in mice gut were highly correlated with CRKP colonization, we calculated LDA scores for each bacteria strain in the microbiome after dividing the microbiomes of the four groups (day5, day7) according to the *K. pneumoniae* level (KP free; KP high loads) obtained from 16S OTU tables. The LEfSe with LDA score findings indicated that *Lactobacillus* was significantly enriched in KP free feces (LDA > 3.0) (**Figure 3F**), while probiotics such as *Bacteroidetes* and *Actinobacteria* were also increased. Therefore, numerous probiotics such as *Lactobacillus, Bacteroidetes*, and *Actinobacteria* were associated with decreased *K. pneumoniae* levels.

### KEGG Pathway Analysis Reveals That *L. plantarum* LP1812 Treatment Increases the Abundance of Fatty Acid Metabolizing Bacteria

To investigate the impact of *L. plantarum* LP1812 on the functions of the mice intestinal microbiome, we annotated our 16S sequences using the KEGG database and identified 274 genes distributed across 42 different pathways (including unclassified pathway, data not shown). There were 13 distinct metabolic pathways (**Figure S2A**), and the abundance changes of metabolism genes indicated that the mice intestinal microbiome of the L. P. group had a significant variation in metabolism from day 0 to day 3 when compared to the other three groups (**Figure S2B**). Additionally, the changes in group clustering demonstrated that the MIX group was considerably closer to L. P. on day 5 and that Blank clustered away from the other three trail groups on the last day. Further analysis of different metabolic pathways indicated that bacteria involved in lipid metabolism were more abundant in the L. P. group which exhibited a greater abundance of lipid metabolism genes than the other three groups from day 1 to day 5, particularly when compared with the Blank (**Figure 3G**). All of these findings suggest that genes involved in lipid metabolism may contribute to the CRKP-anticolonization process.

### Acetic Acid Inhibits *K. pneumoniae In Vitro* by Acidifying Its Intracellular Environment

**Figure 4:**
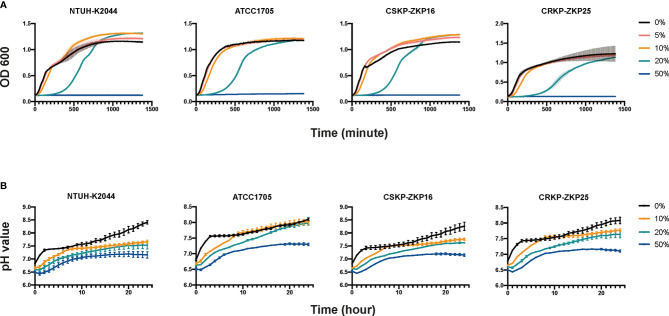
*K. pneumoniae* growth curves and intracellular pH measurements. **(A)** Culturing *K. pneumoniae* in acetic acid-containing LB broth. LB with 50 mmol/L acetic acids (pH=3) is considered as modified LB that is combined with normal LB broth to achieve various concentrations of 5%, 10%, 20%, and 50%. **(B)** Intracellular pH values of *K. pneumoniae*. The aforementioned modified LB broth was combined with normal LB at 10%, 20%, and 50% concentrations.

Because the aforementioned results showed a difference in intestinal lipid metabolism between the trail and blank groups, and given short-chain fatty acids (SCFAs) can help to protect the intestines from pathogenic bacteria colonization (LeBlanc et al., 2017), we hypothesized that SCFAs might play a role in the *L. plantarum* anti-CRKP colonization process. Consequently, the levels of SCFAs in *Klebsiella* spp. and *Lactobacillus* spp. were determined. Acetic acid was the most abundant SCFA in all investigated bacterial strains, with an estimated concentration of 50 mmol/L in *Lactobacillus* spp. (**Table 1**). Hence, we further modified LB broth with 50 mmol/L glacial acetic acid which was equivalent to that in *Lactobacillus* spp. supernatant. The modified LB broth was utilized to replace probiotic supernatant and mixed with normal LB broth as we performed in the assays of **Figure 2**. Those acetic mixtures significantly inhibited *K. pneumoniae* at 20% concentration and weakly at 5% and 10% concentration (**Figure 4A**), which was consistent with probiotic supernatants assays. Acetic acid was shown to be a significant bacteriostat in *Lactobacillus* spp. supernatants.

**Table 1:** The contents of SCFAs in four *Lactobacillus* spp. supernatants (mmol/L).

*L. reuteri* 6110	*L. casei* MLG19_1	*L. plantarum* LP1812	*L. paracaseii *3319
49.36 ± 1.49	46.29 ± 3.14	52.18 ± 3.86	44.28 ± 2.84

Amanda reported that propionic acid may acidify the intracellular environment of *Salmonella* to delay its growth (Jacobson et al., 2018). Therefore, we hypothesized that acetic acid could inhibit *K. pneumoniae* through a mechanism similar to propionic acid. Interestingly, the pH testing results (**Figure 4B**) were consistent with the growth assays results as shown in **Figure 4A**. As 10% and 20% modified LB broth increased the intra-cellular H^+^ ions of *K. pneumoniae*, whist the pH value of *K. pneumoniae* that added with 50% acetic modified LB remained less than 7.5 (**Figure 4B**) which made the bacteria could not proliferate all the time (**Figure 4A**). In summary, the CRKP inhibitory effect of *Lactobacillus* spp. was mediated by acetic acid through acidifying the pathogen’s intracellular environment.

### Anticolonization Effect of Acetic Acid on CRKP Colonization *In Vivo*

**Figure 5:**
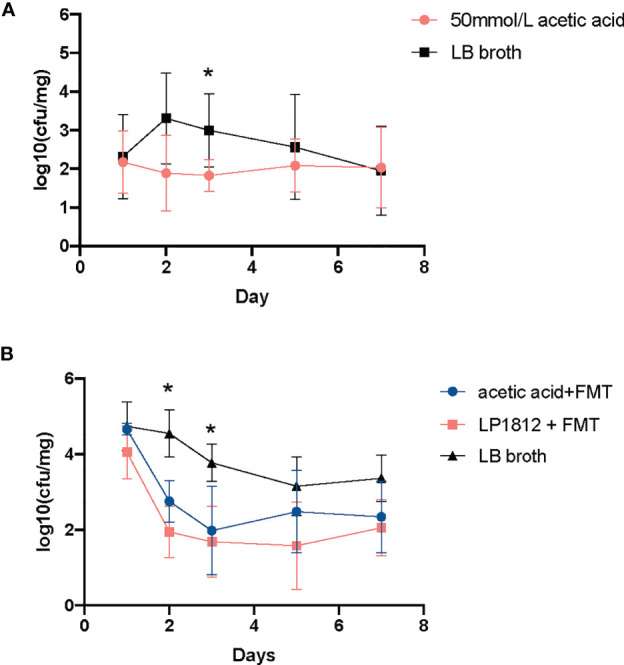
The results of anti-CRKP assays. **(A)** The mice (n = 5) treated with LB broth containing 50 mmol/L acetic acid. **(B)** The mice treated with acetic acid + FMT (n = 5) or *L. plantarum* LP1812 +FMT (n = 5). *denotes the p-value < 0.05.

To further elucidate the role of acetic acid in the anticolonization of CRKP, we repeated the CRKP anti-colonization experiment and replaced *L. plantarum* LP1812 with acetic acid. As shown in **Figure 5A**, acetic acid alone exhibited a significant anti-CRKP colonization effect. More importantly, when combined with FMT, the intestinal protection was significantly enhanced, and the CRKP anticolonization capacity was comparable between acetic acid and *L. plantarum* LP1812 (**Figure 5B**). Therefore, the anticolonization of CRKP by *L. plantarum* LP1812 was mainly mediated by acetic acid.

## Discussion

CRKP belongs to *Enterobacterales*, is regarded as a main nosocomial pathogen that can cause severe infection in critically ill patients (Medrzycka-Dabrowska et al., 2021), and its intestinal colonization is a risk factor for recurrent bloodstream infection (Kontopoulou et al., 2019). Therefore, intestinal clearance of CRKP is important for public health. *Lactobacillus* spp. is generally regarded as safe and has strong pathogen-killing abilities, making them a potential CRKP elimination agent. In the present study, we demonstrated the bactericidal activity of *L. plantarum* LP1812 against CRKP and revealed the critical function of acetic acid in the process of LP1812 clearing CRKP from mice intestines.

The *Lactobacillus* spp. strains used were *L. casei* MLG19_1, *L. reuteri* 6110, *L. paracesei* 3319, as well as *L. plantarum* LP1812. All four strains exhibited a prominent CRKP-killing ability, with *L. plantarum* LP1812 being the most potent. These findings were consistent with the findings of Chi-Chung Chen et al. that *L. plantarum* exhibited a strong pathogen inhibition effect when compared to other *Lactobacillus* spp. (Chen et al., 2019) Mohamed et al. (El-Mokhtar et al., 2020) discovered that this inhibitory effect was mainly achieved by *Lactobacillus* spp. supernatant as we proved in **Figure 2** and *L. plantarum* LP1812 exhibit the strongest CRKP inhibition effect as well. These findings suggested *L. plantarum* LP1812 may be able to alter CRKP intestinal colonization statues.

Although *Lactobacillus* spp. is a potential candidate to eliminate CRKP, the decolonization effect of *Lactobacillus* spp. towards CRKP is not-ideal in clinical trials (Ekmekciu et al., 2017; Ljungquist et al., 2020), which might be due to diverse *Lactobacillus* spp. isolates exert different pathogen-killing and intestinal colonization abilities. We have validated the powerful capacity of *L. plantarum* LP1812 to kill CRKP strains, and the results in **Figure S3B** revealed *L. plantarum* LP1812 can exist in mice intestinal and suffer from pepsin and bile salt, which has been proved by Q Zhou (Zhou et al., 2021) and Mathara (Mathara et al., 2008). In other words, *L. plantarum* LP1812 can normally reproduce and secrete CRKP-killing substances in mice intestinal. More importantly, *L. plantarum* LP1812 indeed decreased the load of CRKP strains in mice feces (**Figure 3B**). Those findings hinted LP1812 might be a potential candidate for utilize in human digestive tract to prevent CRKP colonization.

Additionally, a single inoculation of *L. plantarum* LP1812 can increase the proportions of intestinal probiotics, such as *Bacteroidetes* (Sequeira et al., 2020), *Blautia* (Xia et al., 2021), *Halomanas* (Yu et al., 2021), and *Holdemania* (Raimondi et al., 2021), and the former two can generate acetic acid, which can improve the quantity of SCFAs in the gut (Aoki et al., 2021). Chi-Chung Chen et al. (Chen et al., 2020) reported that acetic acid in the *Lactobacillus* spp. supernatant is a critical factor in mediating CRKP inhibition. Our assays also demonstrated acetic acid’s ability to suppress *K. pneumoniae* growth (**Figure 4A**) and further illustrated the exact CRKP-inhibition mechanism of acetic acid *in vitro*, which was acidifying *K. pneumoniae* intracellular environment (**Figure 4B**). That will damage bacterial vigor and fecundity like Shuichi Nakamura et al. (Nakamura et al., 2009) and Amanda et al. (Jacobson et al., 2018) reported. Although 50 mM acetate used in the *vivo* study is quite a high concentration, the concentration of acetic acid in the lumen of the colon ranges from 10 to 100 mM (Koh et al., 2016), thus acetate in mice intestinal could reach up to the CRKP inhibition concentration (50 mM). Furthermore, acetic acid in the colon can enhance poly-reactive IgA and alter the IgA pool’s ability to bind to *Enterobacterales* (Takeuchi et al., 2021). All of these mechanisms mediated the *in vivo* CRKP inhibition and elimination effect of acetic acid, indicating that acetic acid may contribute to CRKP anticolonization. Our *in vivo* experiments shown in **Figure 5A** confirmed our assumption that acetic acid enhances anti-CRKP intestinal eradication.

Given that, when combined with FMT, LP1812 and acetic acid had equivalent CRKP anti-colonization efficacy (**Figure 5B**), we speculated that acetic acid is a key component in LP1812 that promotes the CRKP elimination process. In other words, the acetic acid in LP1812 that we added to FMT suspensions contributed to alterations in the intestinal microbiota and subsequently achieved CRKP anti-colonization. Unfortunately, we did not perform 16S sequences in acetic acid animal experiments. Further tests are needed to validate this hypothesis.

In conclusion, we found that *L. plantarum* LP1812 can completely eradicate CRKP strains *in vitro* and improve CRKP clearance *in vivo via* altering mice intestinal microbiota. The abundance of bacteria producing acetic acid increased in the colon, ensuring a longer and stronger CRKP-free status. These findings show that *L. plantarum* LP1812 is one of the potential candidates for anticolonization strategies against CRKP infection, which has to be confirmed in clinical practice.

## Data Availability Statement

The datasets presented in this study can be found in online repositories. The names of the repository/repositories and accession number(s) can be found below: https://www.ncbi.nlm.nih.gov/bioproject/; PRJNA776100.

## Ethics Statement

The animal study was reviewed and approved by The Institutional Animal Ethics Committee of Zhejiang University with the number of ZJU20160154.

## Author Contributions

WL provided four *Lactobacillus* spp. strains and YY isolated strains ZKP25 and ZKP16. WL and JZ designed the study and all experiments. RY and YL carried out the assays. XiW, PY, and PL analyzed the data. YR and XuW drafted this manuscript. JY and XP revised the manuscript. All authors have read and approved the submitted version.

## Funding

This work was supported by the Natural Science Foundation of China (No. 81672067, No. 81830069), Project of Zhejiang Bureau of Traditional Chinese Medicine (No. 2022ZB375), Hangzhou Agricultural and Society Development Project (grant No. 202004A20), and Zhejiang Public welfare project (LGN19C010003).

## Conflict of Interest

The authors declare that the research was conducted in the absence of any commercial or financial relationships that could be construed as a potential conflict of interest.

## Publisher’s Note

All claims expressed in this article are solely those of the authors and do not necessarily represent those of their affiliated organizations, or those of the publisher, the editors and the reviewers. Any product that may be evaluated in this article, or claim that may be made by its manufacturer, is not guaranteed or endorsed by the publisher.
