# Supplementary material for: Anticolonization of Carbapenem-Resistant Klebsiella pneumoniae by Lactobacillus plantarum LP1812 Through Accumulated Acetic Acid in Mice Intestinal
Source: Front Cell Infect Microbiol. 2021 Dec 15;11:804253. doi: 10.3389/fcimb.2021.804253 (PMC8714838; doi:10.3389/fcimb.2021.804253)
Supplement: Supplementary file 1 [file DataSheet_1.zip › SupplementaryMateria/Supplementary figure legends.docx]

**Supplementary Figure 1.** PCA analysis based on OTU tables of the four groups, which is blank control and test groups: L.P., FMT, MIX. Among test groups, FMT was regarded as positive control.

**Supplementary Figure 2.** The results of 16S KEGG analysis for metabolism pathways. (B) Clustered the four groups in each timepoints, day -4, 0, 1, 3, 5 and 7. High loads was colored as red and blue squares mean the low loads of species that highly associated with relative functions. And the exact meanings of M1 to M11 was listed in the left panel (A).

**Supplementary Figure 3.** (A)The $\alpha$-diversity of feces microbiomes. (B) The loads of L. plantarum in mice feces, that data came from 16S OTU tables.
