# Supplementary figures and images for: Anticolonization of Carbapenem-Resistant Klebsiella pneumoniae by Lactobacillus plantarum LP1812 Through Accumulated Acetic Acid in Mice Intestinal
Source: Front Cell Infect Microbiol. 2021 Dec 15;11:804253. doi: 10.3389/fcimb.2021.804253 (PMC8714838; doi:10.3389/fcimb.2021.804253)

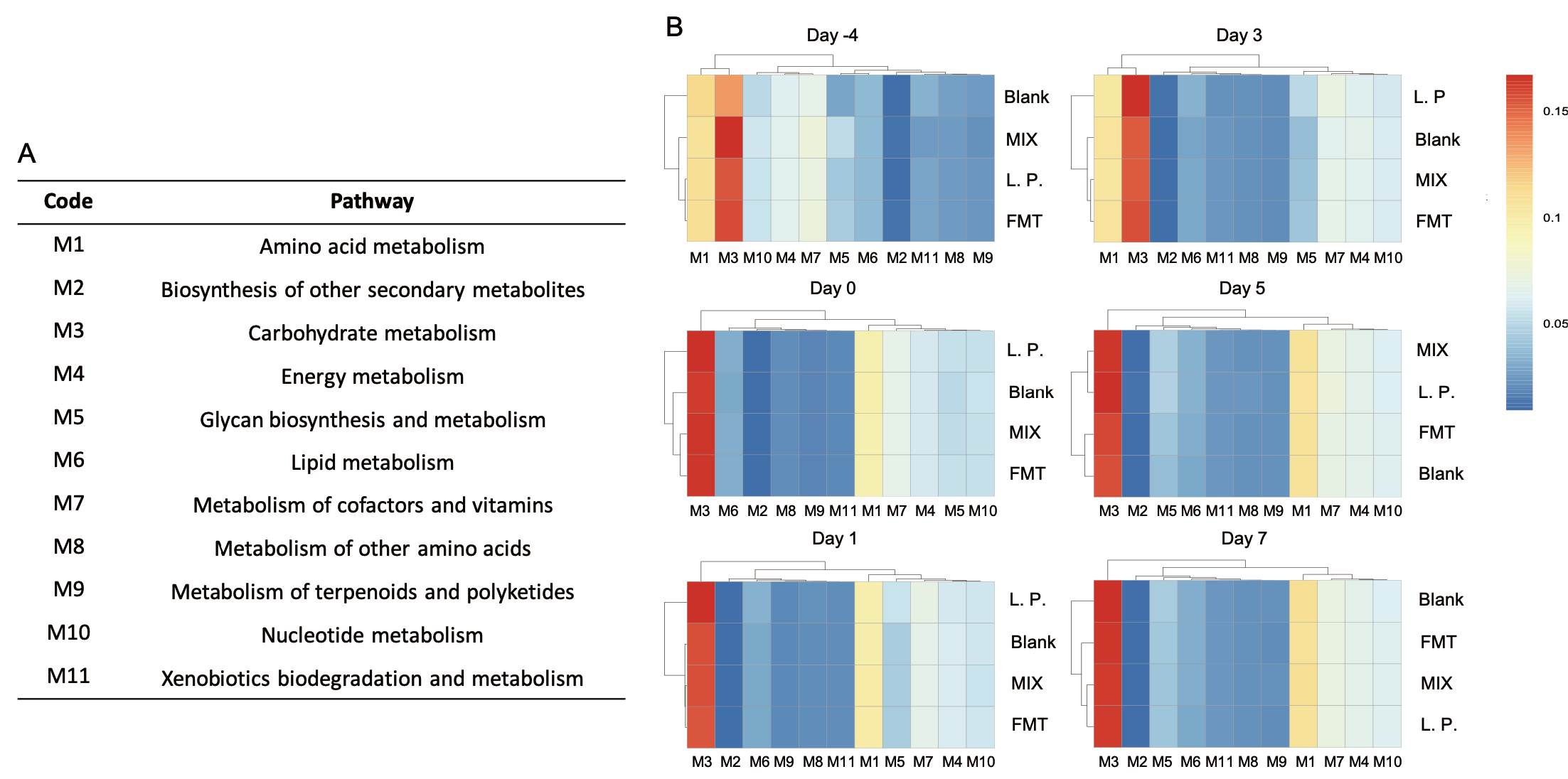

Supplement: Supplementary file 1 [file DataSheet_1.zip › SupplementaryMateria/SupFigure2.tiff]

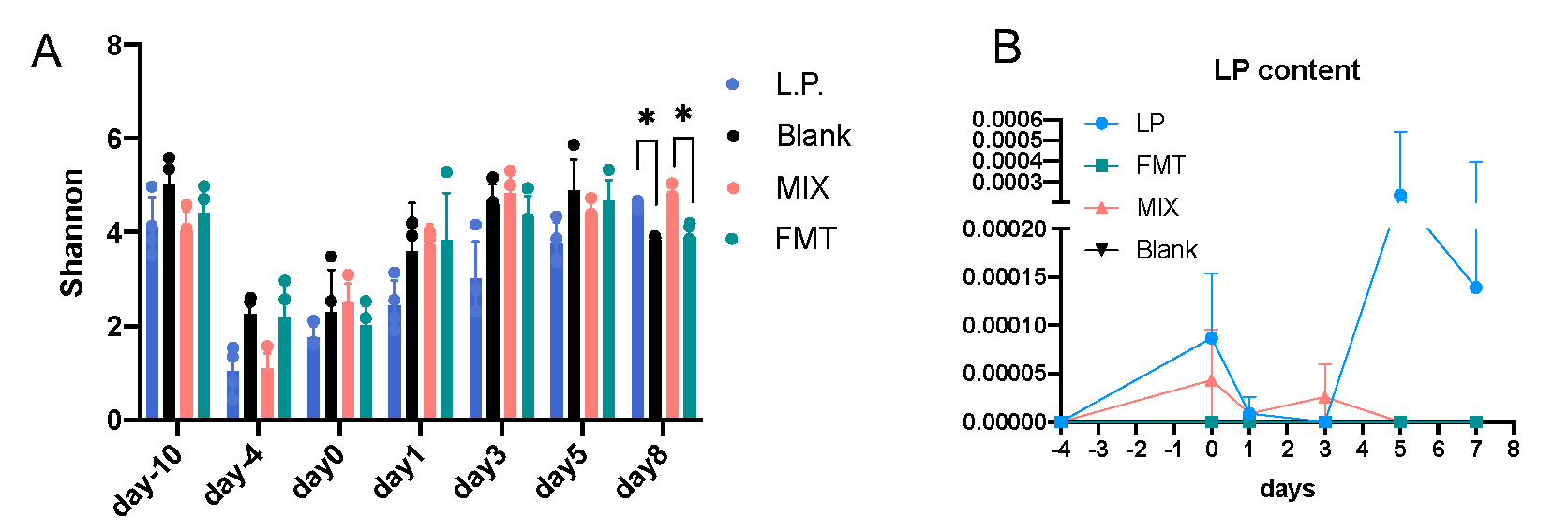

Supplement: Supplementary file 1 [file DataSheet_1.zip › SupplementaryMateria/SupFigure3.tiff]

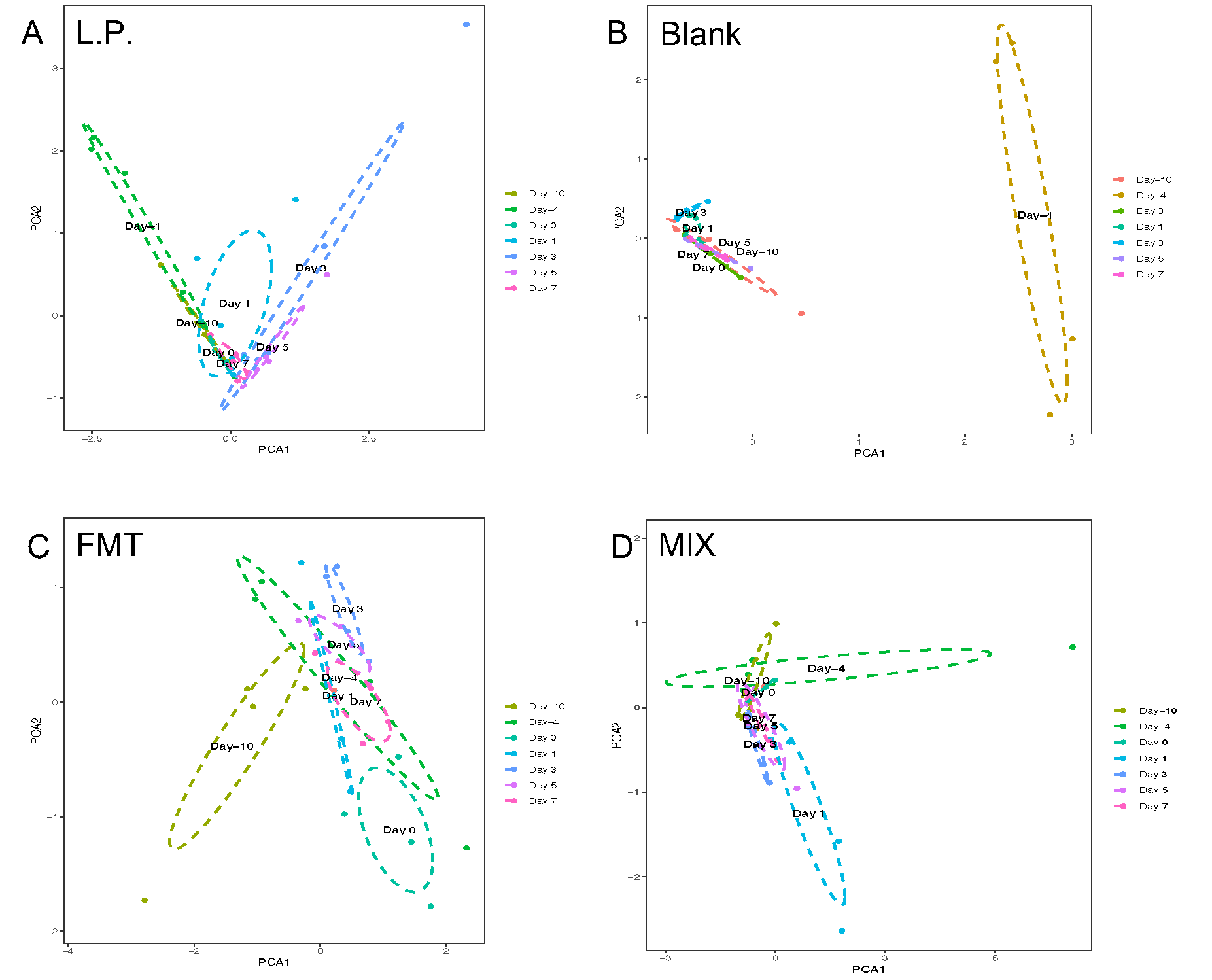

Supplement: Supplementary file 1 [file DataSheet_1.zip › SupplementaryMateria/SupFigure1.tiff]
